# De novo deoxyribonucleotide biosynthesis regulates cell growth and tumor progression in small-cell lung carcinoma

**DOI:** 10.1038/s41598-021-92948-9

**Published:** 2021-06-29

**Authors:** Ami Maruyama, Yuzo Sato, Joji Nakayama, Junko Murai, Takamasa Ishikawa, Tomoyoshi Soga, Hideki Makinoshima

**Affiliations:** 1 Shonai Regional Industry Promotion Center, Tsuruoka, Yamagata 997-0052 Japan; 2 Tsuruoka Metabolomics Laboratory, National Cancer Center, Mizukami 246-2, Kakuganji, Tsuruoka, Yamagata 997-0052 Japan; 3 Division of Translational Research, Exploratory Oncology Research, and Clinical Trial Center, National Cancer Center, Kashiwa, Chiba 277-8577 Japan; 4 Systems Biology Program, Graduate School of Media and Governance, Keio University, Fujisawa, 252-0882 Japan; 5 Institute for Advanced Biosciences, Keio University, Tsuruoka, 997-0035 Japan; 6 Infinity Lab Inc., Mizukami 246-2, Kakuganji, Tsuruoka, Yamagata 997-0052 Japan; 7 Faculty of Medicine, Yamagata University, Yamagata, 990-9585 Japan

**Keywords:** Lung cancer, Metabolomics

## Abstract

Deoxyribonucleotide biosynthesis from ribonucleotides supports the growth of active cancer cells by producing building blocks for DNA. Although ribonucleotide reductase (RNR) is known to catalyze the rate-limiting step of de novo deoxyribonucleotide triphosphate (dNTP) synthesis, the biological function of the RNR large subunit (RRM1) in small-cell lung carcinoma (SCLC) remains unclear. In this study, we established siRNA-transfected SCLC cell lines to investigate the anticancer effect of silencing *RRM1* gene expression. We found that RRM1 is required for the full growth of SCLC cells both in vitro and in vivo. In particular, the deletion of RRM1 induced a DNA damage response in SCLC cells and decreased the number of cells with S phase cell cycle arrest. We also elucidated the overall changes in the metabolic profile of SCLC cells caused by RRM1 deletion. Together, our findings reveal a relationship between the deoxyribonucleotide biosynthesis axis and key metabolic changes in SCLC, which may indicate a possible link between tumor growth and the regulation of deoxyribonucleotide metabolism in SCLC.

## Introduction

Small-cell lung carcinoma (SCLC) is a neuroendocrine tumor subtype of lung cancer, and it is associated with a poor prognosis^1–3^. In particular, SCLC is characterized by early and widespread metastatic dissemination and a remarkable response to chemotherapy that is almost invariably followed by the development of drug resistance^4^. The first-line therapy for patients with SCLC has not changed for several decades; however, studies have recently shown that lurbinectedin could be an effective second-line therapy^5^. Although it has been shown that SCLC has a high frequency of *TP53* and *RB1* mutations, these gene mutations are not effective drug targets^3,6^. As a result of these limited therapeutic options, novel targeted molecular agents are urgently required for SCLC. In recent years, research has particularly aimed to develop new therapies to address the problem of treatment resistance in SCLC and the associated vulnerabilities^7–9^.

The metabolic features of SCLC are nucleic acid biosynthesis and degradation pathways^10–12^. Deoxyribonucleotides are DNA building blocks that are essential for accurate DNA replication and repair; therefore, an adequate and balanced supply of deoxynucleotides is crucial for maintaining genome stability^13–16^. Two distinct deoxyribonucleotide triphosphate (dNTP) biosynthesis pathways exist in eukaryotic cells: (1) de novo dNTP synthesis in the cytosol; and (2) the mitochondrial deoxynucleotide salvage pathway, which maintains the mitochondrial dNTP pool throughout the cell cycle^17–20^. De novo dNTP synthesis is associated with the cell cycle and supplies the most deoxynucleotides during S phase for the replication of genomic DNA^17,18^. The de novo biosynthesis of deoxycytidine triphosphate (dCTP), deoxyadenosine triphosphate (dATP), and deoxyguanosine triphosphate (dGTP) is highly dependent on the activity of ribonucleotide reductase (RNR, Fig. S1), which catalyzes the rate-limiting step in dNTP synthesis and is a well-recognized target for cancer therapy^20,21^. Importantly, studies using clinical specimens, cell lines, and mouse models have recently revealed characteristic metabolic regulation in SCLC, including nucleic acid metabolism^10–12,22^. Therefore, we hypothesized that nucleic acid biosynthesis pathways could play pivotal roles in SCLC and represent feasible drug targets.

RNR is a heterotetramer consisting of two large RRM1 subunits and two small RRM2 and RRM2B (p53R2) subunits that catalyze the reduction of ribonucleotide diphosphates (NDPs) into their corresponding deoxyribonucleotides (dNDPs)^21,23^. The ratio of ribonucleotides to dNDPs is crucially maintained by multiple steps that tightly regulate RNR^18,21,23^. RRM1 is a DNA repair gene, and the expression of RRM1 is correlated with the response to chemotherapy and survival of patients with lung cancer^24–26^. There is already a collection of papers in the literature on the effects on cell proliferation and survival in cancer cells following inhibition of RRM1 expression and function^27–30^. However, the biological role of RRM1 in SCLC was not addressed until now. The first mechanism to be discovered that regulates RNR enzyme activity involved allosteric control, in which the transcriptional activation of RRM2 during the S/G2 phase greatly stimulates RNR activity to ensure adequate dNTP supply for DNA replication^18,21,23^. Recently, several RNR protein regulatory mechanisms have been reported involving post-translational modification^31,32^; however, little is known about the biological role of RRM1 in SCLC.

Here, we demonstrate that RRM1 is required for the full growth of SCLC cells both in vitro and in vivo, and determined the changes in the metabolic profile of SCLC cells following RRM1 deletion.

## Results

**Figure 1:**
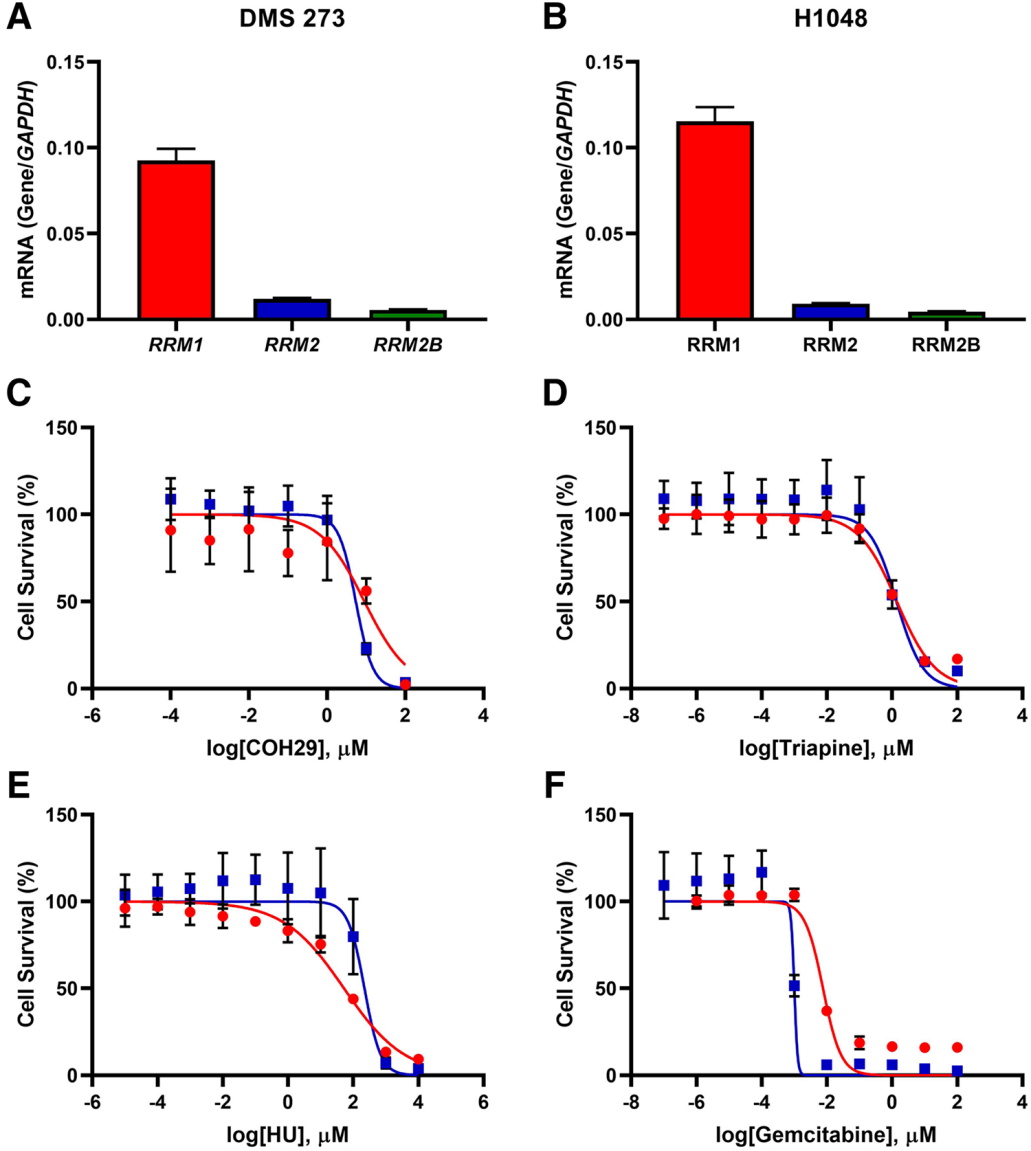
RRM1 expression and sensitivity to RRM1 inhibitors. *A* and *B*, mRNA expression of RNR-related genes in DMS 273 (**A**) and NCI-H1048 (**B**) cells. *RRM1* (red)*, RRM2* (blue), and *RRM2B* (green) levels were normalized to glyceraldehyde 3‐phosphate dehydrogenase (*GAPDH*) expression. Data represent the mean ± standard deviation (SD; *n* = 3). (**C**–**F**), WST-8 assay with the RRM1 inhibitors COH29 (*C*), triapine (**D**), hydroxyurea (**E**), and gemcitabine (**F**). Cells were treated with the indicated inhibitors for 3 days and their viability was assessed using the WST-8 assay. Red circle: DMS 273, blue square: H1048. Data represent the mean ± SD (*n* = 6–10).

To verify whether RRM1, the main enzyme in RNR, was expressed in SCLC cell lines, we measured *RRM1* gene expression in SCLC cells using RT-PCR (Fig. S2). Although differences in *RRM1* mRNA expression were observed among cell lines, COR-L32 displayed the highest expression (*RRM1/GAPDH* = 0.386) and COR-L88 displayed the lowest (*RRM1/ GAPDH* = 0.044; Fig. S2). To confirm these findings, we also measured the expression of RNR-related proteins (RRM1, RRM2, and RRM2B) in SCLC cell lines using western blotting (Fig. S3). Consequently, we used two SCLC cell lines, DMS 273 (hereinafter abbreviated to 273) and NCI-H1048 (hereinafter abbreviated to H1048), for all subsequent experiments as both expressed the *RRM1* gene at much higher levels than *RRM2* and *RRM2B* (Fig. 1A,B).

Because RNR inhibitors are used in some clinics and clinical trials, we investigated the susceptibility of 273 and H1048 cells to these drugs by evaluating the 50% inhibitory concentration (IC_50_), which is often used to determine drug potency with cell-based cytotoxicity tests. The IC_50_ values for the RRM inhibitors COH29, triapine, hydroxyurea (HU), and gemcitabine were 9.04, 52.6, 1.41, and 0.00763 μM in 273 cells and 5.44, 223, 1.37, and 0.00101 μM in H1048 cells, respectively (Fig. 1C–F). Although the IC_50_ values for COH29, triapine, and HU were relatively high, gemcitabine was able to effectively repress the growth of SCLC cells. This may be because triphosphate gemcitabine forms also inhibit DNA polymerases by incorporation into DNA^33,34^.

Because RRM1 mRNA was expressed in all SCLC cell lines (Fig. S2), we characterized the role of RRM1 in cell growth and deoxyribonucleotide biosynthesis in SCLC cells by employing a genetic RNA interference (RNAi) approach to repress *RRM1* expression. Three different siRNA sequences directed against *RRM1* (si*RRM1*#1, #2, and #3) and a non-targeting siRNA (siCont) were used to combat potential off-target siRNA effects.

**Figure 2:**
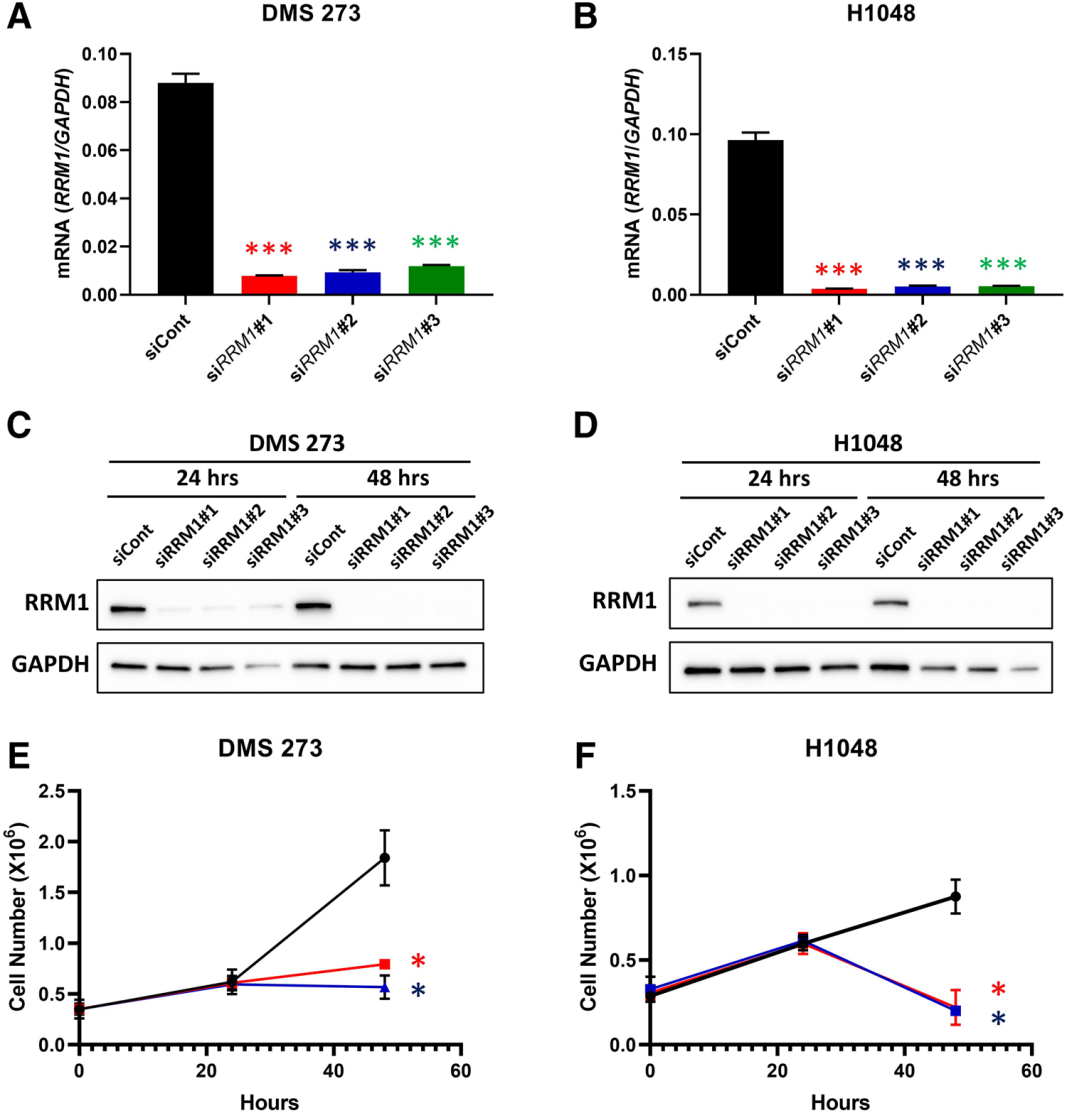
*RRM1* knockdown in SCLC cell lines. (**A**) and (**B**), effect of *RRM1*-siRNA on *RRM1* mRNA levels. *RRM1*-specific siRNAs (si*RRM1*#1: red, si*RRM1*#2: blue, si*RRM1*#3: green, siCont: black) were used to knock down *RRM1* in SCLC cells. *RRM1* expression was normalized to that of *GAPDH*. Data represent the mean ± standard deviation (SD; *n* = 3). *P* values are indicated as ***, < 0.005, Dunnett’s multiple comparisons test. (**C**) and (**D**), effect of *RRM1*-siRNA on RRM1 protein levels. Western blot analysis of si*RRM1*‐treated SCLC cells with GAPDH as the loading control. The blots were cropped; full-length blots are included in the Supplementary Information. (**E**) and (**F**), cell growth inhibition measured using the proliferation assay. siCont: black, si*RRM1*#1: red, si*RRM1*#3: blue. Data represent the mean ± SD (*n* = 6–10). *p < 0.05 *vs.* control, Dunnett’s multiple comparisons test.

Quantitative RT-PCR revealed that si*RRM1* significantly downregulated *RRM1* mRNA expression by 85% in 273 cells (Fig. 2A) and 90% in H1048 cells (Fig. 2B) compared with control siCont. Immunoblot analyses also revealed significant decreases in RRM1 protein expression in 273 (Fig. 2C) and H1048 (Fig. 2D) cells under normal culture conditions following the introduction of si*RRM1*#1, #2, and #3 compared with siCont. We also measured the growth of SCLC cells in vitro following RRM1 knockdown, finding that there were significantly fewer RRM1-knockout 273 and H1048 cells compared with control cells (Fig. 2E,F), suggesting that RRM1 deletion significantly reduces the proliferation of SCLC cells.

Because the loss of RRM1 expression in vitro inhibited SCLC cell growth, we investigated the role of RRM1 in tumor growth in vivo using an immunodeficient nude (*nu/nu*) murine xenograft model subcutaneously transplanted with human SCLC cells with in vivo siRNA delivery. Briefly, the flanks of nude mice (*n* = 3 or 4) were subcutaneously injected with 5 × 10^6^ SCLC cells suspended in 200 μL of RPMI-1640 medium and tumor size was measured over time. Because siRNA only suppresses gene expression temporarily, we introduced siRNA (siCont and si*RRM1*, 2 nmol/injection with AteloGene) before transplantation, after tumor formation, and every 3 days.

**Figure 3:**
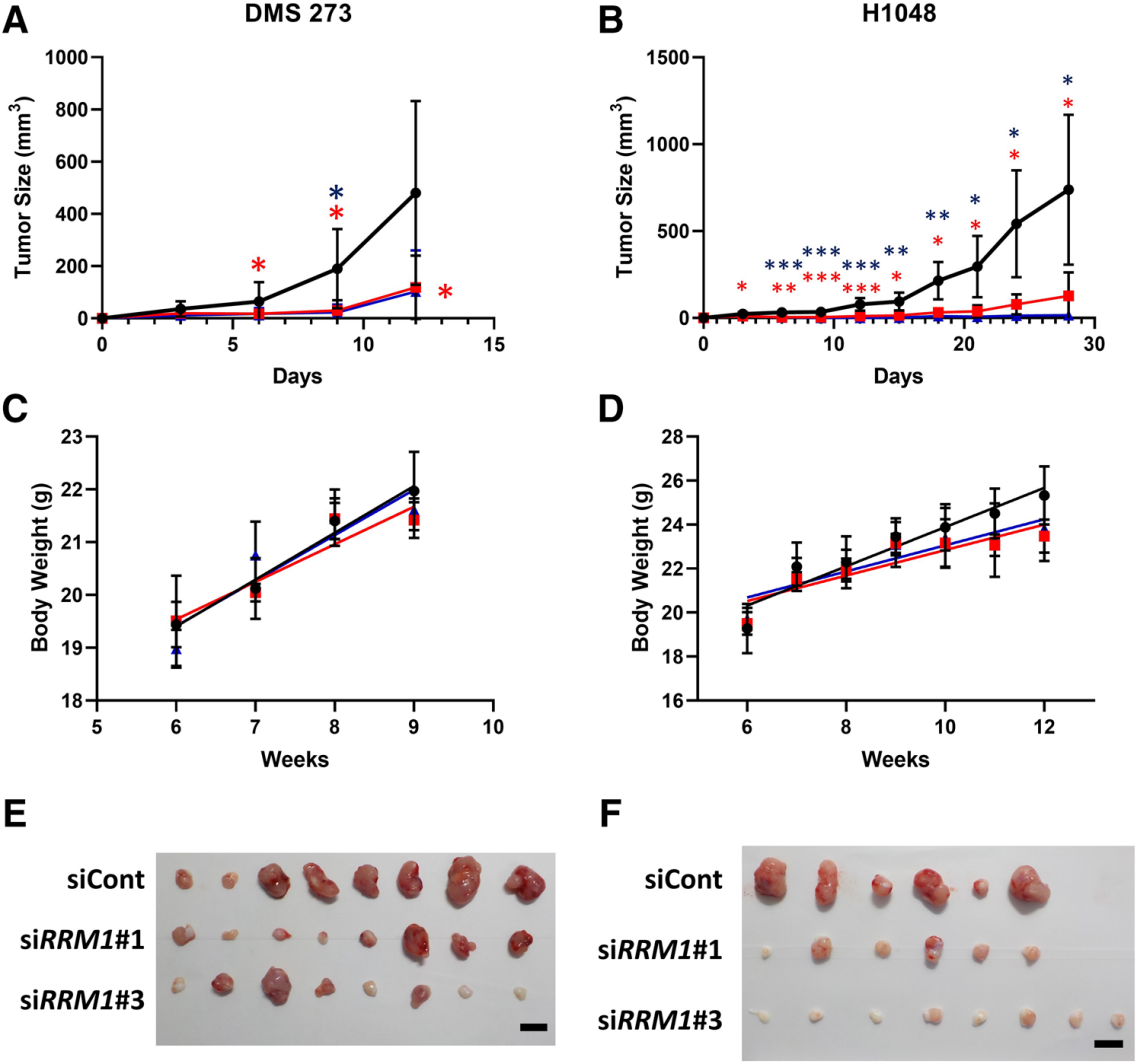
RRM1 regulates tumor progression in an SCLC xenograft mouse model. (**A**) and (**B**), in vivo growth of SCLC DMS 273 (**A**) and H1048 (**B**) cell tumors in nude mice transfected and injected with siRNA every 3 days (*n* = 6–8). siCont: black, si*RRM1*#1: red, si*RRM1*#3: blue. *p < 0.05; **p < 0.01, ***p < 0.005 *vs.* control, Dunnett’s multiple comparisons test. *C* and *D*, body weight of mice transplanted with 273 (**C**) and H1048 cells (**D**). siCont: black, si*RRM1*#1: red, si*RRM1*#3: blue. Data represent the mean ± SD (*n* = 3–4). *E* and *F*, gross tumor pathology in tumors derived from 273 (**E**) and H1048 cells (**F**) at the end of the experiments. Scale bars = 10 mm.

In the mouse model, 273 + siCont cells started to grow exponentially 6 days post-transplant and progressively formed tumor masses (Fig. 3A); however, tumor formation by the 273 + si*RRM1* cells was impaired until 12 days post-transplant, such that tumors were barely visible under the skin and were approximately fourfold smaller than those formed from 273 + siCont cells (Fig. 3A). Similarly, RRM1 knockout in H1048 cells decreased the rate of tumor formation in nude mice compared with cells expressing the siCont control (Fig. 3B). We also measured the body weight of the mice to rule out any side effects of siRNA injection and AteloGene, observing no weight loss until the end of the in vivo experiments (Fig. 3C,D). Gross examination of both 273 and H1048 + si*RRM1* tumors revealed a dramatic loss of SCLC pathology (Fig. 3E,F), whereas the RRM1 expression was confirmed in the formed tumor (Fig. S4). The histological characterization of the SCLC xenografts is shown in Supplementary Figure S5. Together, these results suggest that the RRM1 protein is essential for the formation of SCLC tumors.

**Figure 4:**
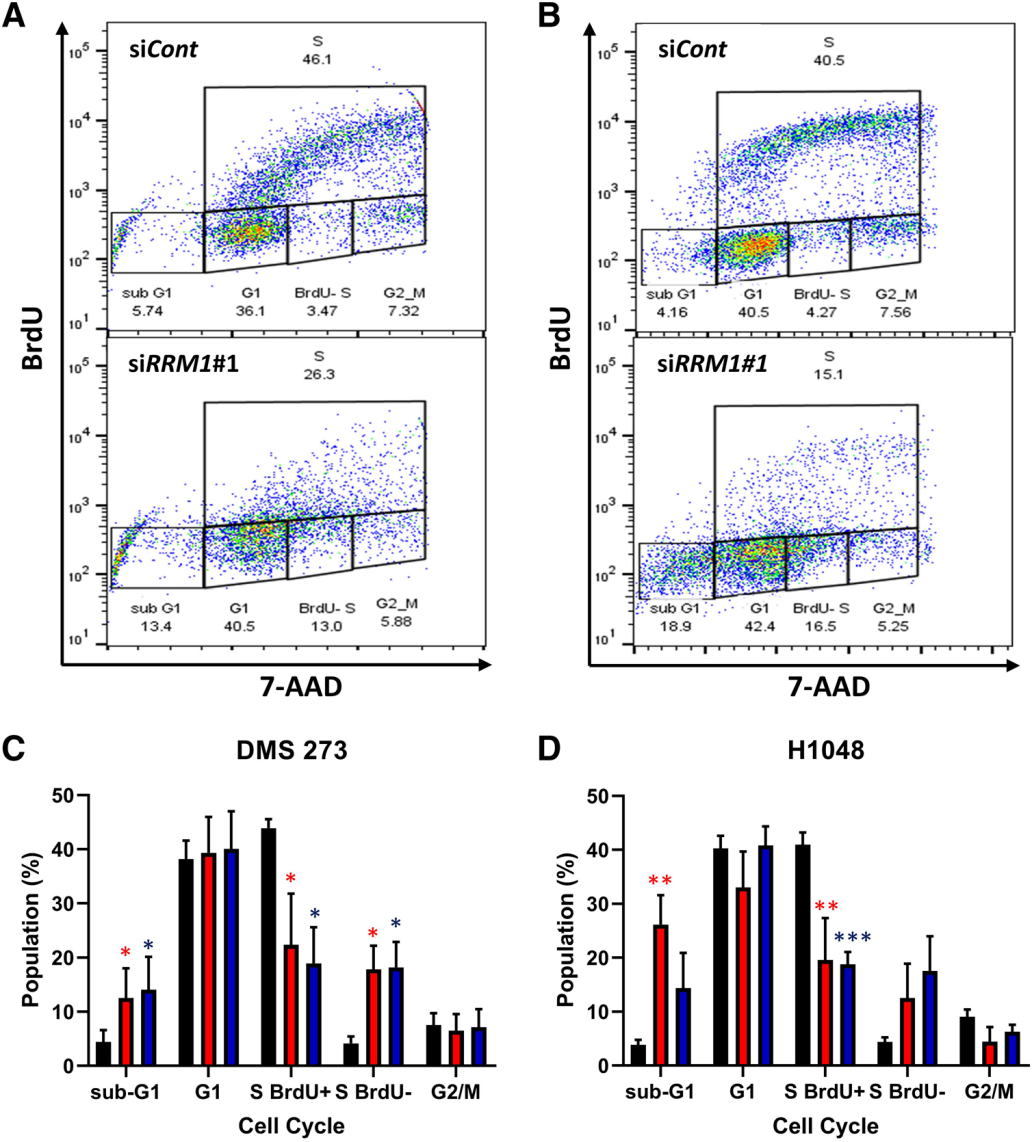
RRM1 knockout stalls DNA replication and induces cell cycle arrest. (**A**) and (**B**), representative flow cytometry cell cycle data for siRNA-treated 273 (**A**) and H1048 **(B)** cells after double staining for BrdU incorporation (S phase cells) and 7-AAD (DNA content). (**C**) and (**D**), cell cycle analysis of DMS 273 (**C**) and H1048 (**D**) SCLC cells transfected with si*RRM1* showing the percentage of cells in sub-G1, G1, S (BrdU −), S (BrdU +), and G2/M phase. Black: siCont, red: si*RRM1*#1, blue: si*RRM1*#3. Data represent the mean ± SD (*n* = 4). *p < 0.05, **p < 0.01, ***p < 0.005 *vs.* control, Dunnett’s multiple comparisons test.

Imbalances in the dNTP pool can be deleterious and lead to frameshift errors, replication stress, and G1/S arrest^18,21,23^. To elucidate the relationship between RRM1-mediated DNA biosynthesis and cell proliferation, we analyzed the cell cycle in the presence or absence of RRM1. Because dNTP production for DNA biosynthesis is often the highest during the S phase of the cell cycle^21,23^, we performed double staining flow cytometry analysis with 5-bromo-2’-deoxyuridine (BrdU) to evaluate S phase cells and 7-amino-actinomycin D (7-AAD) to measure DNA content. The number of cells and newly biosynthesized DNA content were lower when treated with si*RRM1* for 48 h, as indicated by the number of BrdU positive (+) cells (Fig. 4A,B). The number of sub-G1 cells treated with si*RRM1*#1 was increased, at 12.5 ± 5.48% or 26.2 ± 5.46% of the SCLC cell population, compared with 4.44 ± 2.18 or 3.84 ± 0.944% with siCont in 273 or H1048 cells, respectively (Fig. 4C,D). The number of replicating S-phase cells was dramatically decreased in both 273 and H1048 cells treated with si*RRM1* for 48 h (Fig. 4C,D). Treatment with si*RRM1* also increased the number of nonreplicating S-phase cells that were BrdU-negative at 1.78 ± 4.34% or 12.5 ± 6.37% of the SCLC cell population, compared with 4.15 ± 1.27% or 4.41 ± 0.812% with siCont in both 273 and H1048 cells, respectively (Fig. 4C,D). These results indicate that the loss of RRM1 inhibits DNA replication during S phase in 273 and H1048 cells.

**Figure 5:**
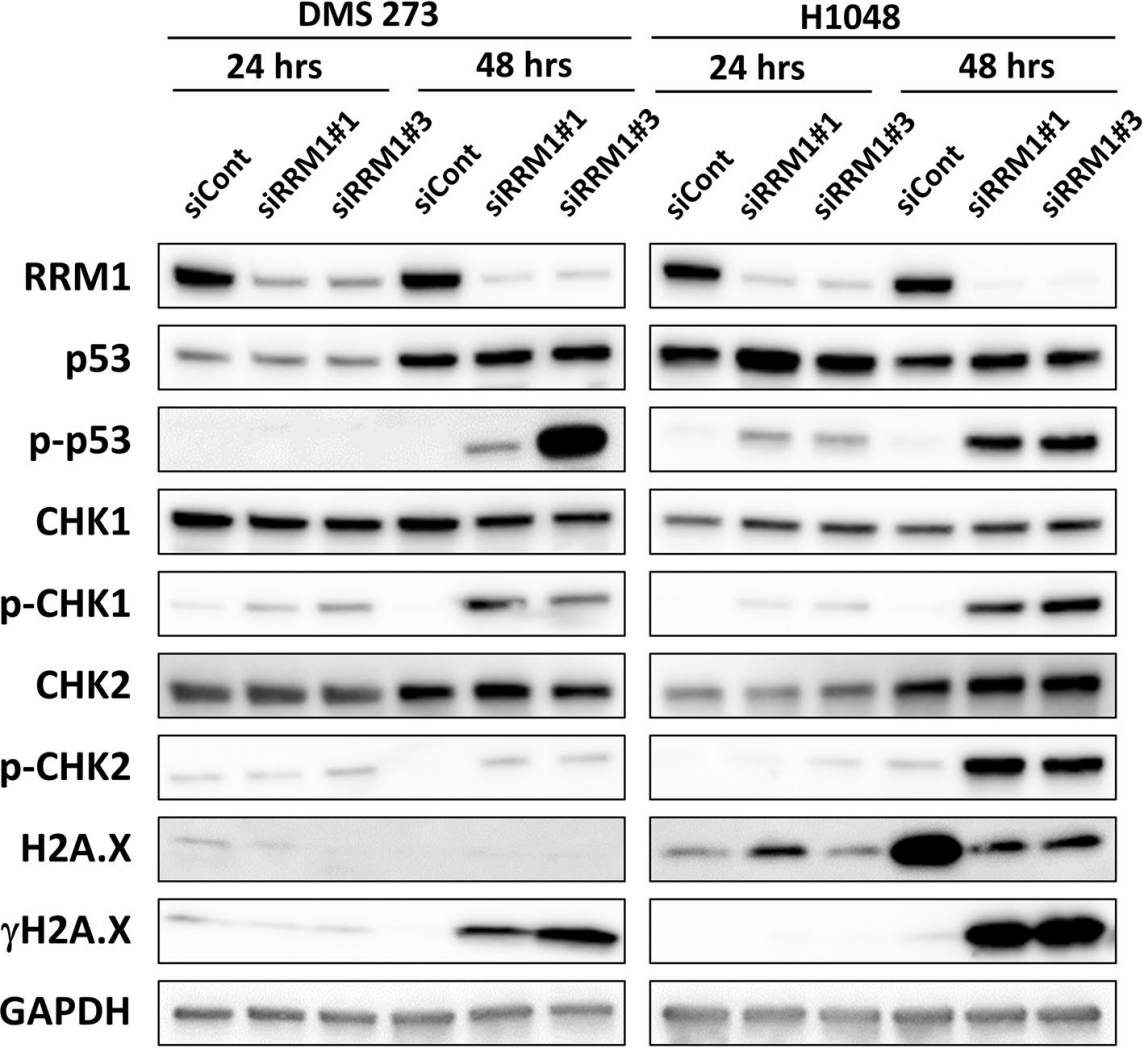
Representative immunoblot analysis of DNA damage-related proteins in RRM1-knockdown SCLC cells. siRNA-transfected SCLC cells were subjected to western blotting at the indicated time. Total proteins and phosphorylated (p-) proteins were detected using specific antibodies: RRM1, p53, p-p53 (S15), CHK1, p-CHK1 (S345), CHK2, p-CHK2 (T68), H2A.X, and γH2A.X (S139), with GAPDH as the loading control. The blots were cropped, and full-length blots are included in the Supplementary Information.

Imbalance in the dNTP pool has been associated with the DNA dameg response (DDR), whereas links between DNA replication and transcription may underlie DNA replication stress and limit the dNTP pool^18,21,23^; therefore, RRM1 knockdown may affect the DDR and/or repair genes. To confirm whether RRM1 knockdown affected DDR signaling in SCLC cells, we performed western blot analysis in siRRM1‐treated SCLC cells at the indicated time points. As expected, RRM1 expression was successfully knocked down, whereas total p53 protein levels were elevated in H1048 cells and phosphorylated p53 (p-p53) was upregulated in both 273 and H1048 cells 48 h after siRNA introduction (Fig. 5). Total CHK1 (checkpoint kinase 1) and CHK2 (checkpoint kinase 2) protein levels were comparable in 273 and H1048 cells treated with both siCont and siRRM1; however, phosphorylated CHK1 (p-CHK1) and CHK2 (p-CHK2) levels increased 48 h after siRRM1 transfection (Fig. 5). Similarly, total H2A.X levels were comparable, but γH2A.X levels increased 48 h after RRM1 knockout in both 273 and H1048 cells. Densitometric analysis of all the immunoblots and associated data are shown as Supplementary Table S1. Together, these findings demonstrate that RRM1 knockdown triggers the DDR in SCLC cells.

**Figure 6:**
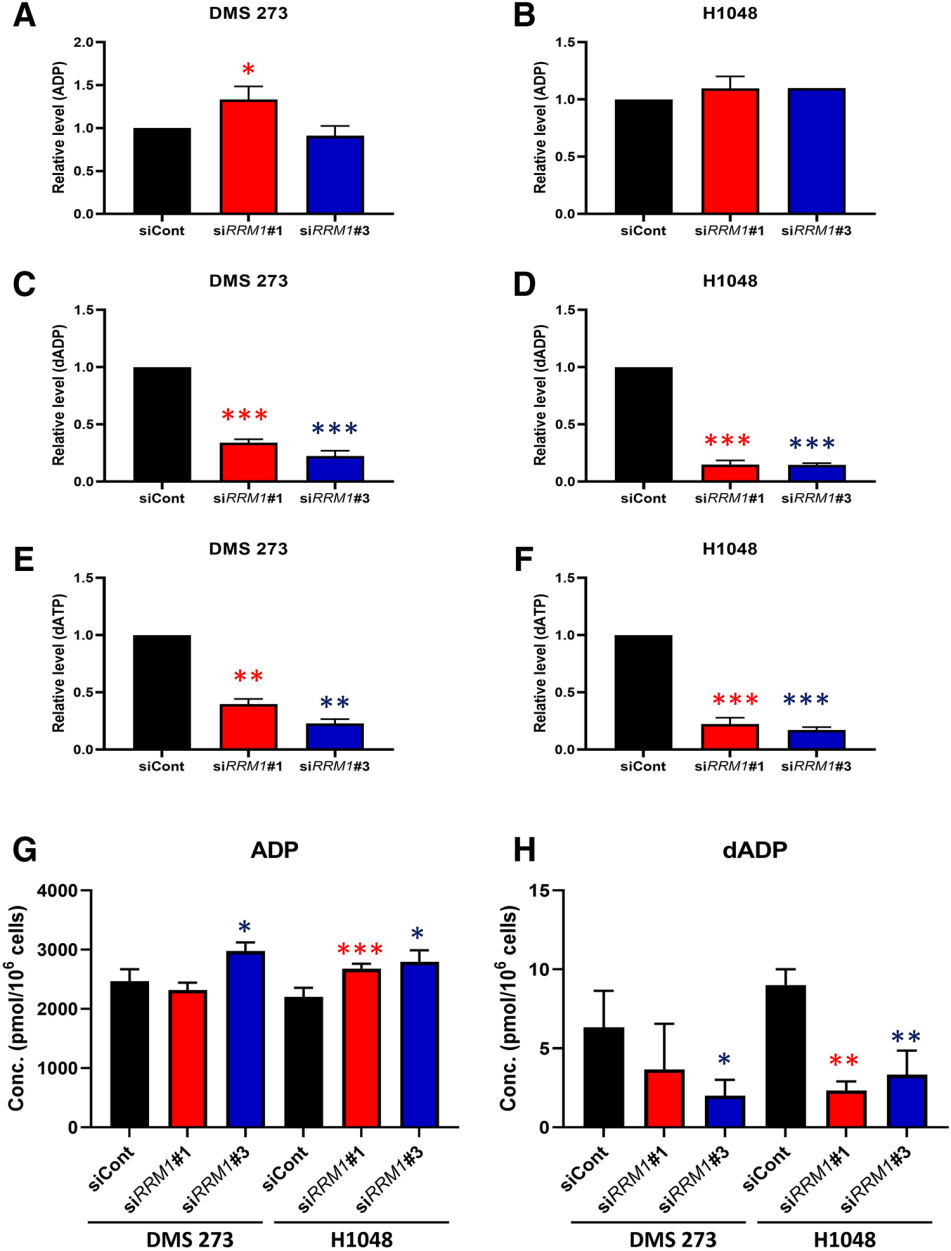
RRM1 knockout decreased dNTP levels in SCLC cells. *A*–*F*, relative intracellular levels of key metabolites in the nucleotide biosynthesis pathway after RRM1 inhibition. The representative metabolites ADP, dADP, dATP, and ATP are shown here; however, others are listed in Supplementary Table S2. Data represent the mean ± SD (*n* = 3). Black: siCont, red: si*RRM1*#1, blue: si*RRM1*#3. Welch *t*-test and *p* values are indicated as *, < 0.05; **, < 0.01; and ***, < 0.001. *G* and *H*, intracellular concentrations (pmol/10^6^ cells) of ADP and dADP after RRM1 inhibition in DMS 273 (*G*) and H1048 (*H*) cells. Data represent the mean ± SD (*n* = 3). Black: siCont, red: si*RRM1*#1, blue: si*RRM1*#3. *p < 0.05, **p < 0.01, ***p < 0.001 *vs.* control, Welch *t*-test.

To determine whether the loss of RRM1 affects global cancer metabolism, we performed metabolome analysis using the ω*-Scan* package from Human Metabolome Technologies (HMT) Inc. (Tsuruoka, Yamagata, Japan) and a capillary electrophoresis Orbitrap mass spectrometer (CE-FTMS), as described previously^35–38^. Intracellular metabolites, including RRM dNDP substrates (adenosine diphosphate [ADP], guanosine diphosphate [GDP], cytidine diphosphate [CDP], and uridine diphosphate [UDP]) and dNTP products (Fig. S1), were extracted using methanol and analyzed using capillary electrophoresis time-of-flight mass spectrometry. The profile of 427 quantified metabolites is shown in Supplementary Table S2 and the representative results of ADP are shown in Fig. 6. While ADP levels were comparable in 273 and H1048 cells with or without RRM1 knockdown (Fig. 6A,B), the introduction of siRRM1 dramatically decreased deoxy-ADP (dADP) levels in both 273 and H1048 cells (Fig. 6C,D), as well as levels of the downstream metabolite, dATP. To confirm these metabolomic findings, we quantified ADP and dADP using high-performance liquid chromatography (HPLC). Consistently, ADP levels were comparable, and dADP levels were reduced after RRM1 knockdown in SCLC cells (Fig. 6G,H). We demonstrate that the deletion of RRM1 reduces the quantity of metabolites produced by RRM1, and we also measured the reduction in the quantity of metabolites present in the downstream metabolic pathway.

## Discussion

SCLC is associated with a poor prognosis due to metastatic dissemination, drug resistance, and a lack of therapeutic targets; however, recent studies have demonstrated that SCLC displays characteristic abnormalities in nucleic acid metabolism. RNR catalyzes the rate-limiting step in dNTP synthesis and is a well-recognized target for cancer therapy; however, the biological role of RRM1, the main catalytic subunit of RNR, in SCLC remains unclear. In this study, we demonstrated that RRM1 is required for the full growth of SCLC cells in vitro and in vivo, with RRM1 knockdown activating DDR signaling and increasing the number of cells in cell cycle arrest. Moreover, we revealed the overall changes in the metabolic profile of SCLC cells caused by RRM1 deletion. Taken together, our findings suggest that RRM1 could be a potential target for the development of new molecular targeting therapies for SCLC, whereas the metabolite profile could be used as a biomarker for assessing the sensitivity and response to RRM1 therapy.

The biological function of RRM1 in lung cancer is controversial and may require further research. Overexpression of RRM1 in non-small-cell carcinoma (NSCLC) cells induced PTEN expression and suppressed metastasis^39^. Furthermore, functional analysis based on a cell biological approach suggested that the RRM1 gene is a tumor suppressor^40^. A characteristic feature of SCLC in terms of pathology is the high rate of mitosis, apoptosis, and necrosis^41,42^. Due to the vigorous cell division of SCLC, the demand for biosynthesis of nucleic acids such dNTPs might be higher than in other cells. In this paper, we showed that RRM1 has an important function in the growth of SCLC, but how much it contributes to the development of SCLC tumors, including metastasis, is a topic that should be addressed in the future.

RNR activity is initiated by the generation of a free radical following a single reduction, which requires electrons donated from the dithiol groups of the protein thioredoxin^21,23^. To regenerate the dithiol groups on thioredoxin, nicotinamide adenine dinucleotide phosphate (NADPH) provides two hydrogen atoms to reduce its disulfide groups^21,23^. However, we detected no statistically significant differences in the levels of antioxidants such as glutathione (GSH), divalent glutathione (GSSG), nicotinamide adenine dinucleotide, or NADPH in either 273 or H1048 cells (Fig. S6).

Although previous studies have investigated RRM1 expression in SCLC specimens to predict the efficacy of DNA biosynthesis inhibitors, such as cisplatin, carboplatin, etoposide, and gemcitabine^24–26^, the biological role of RRM1 in SCLC has not been addressed until now. The findings of our study demonstrate a novel relationship between the deoxyribonucleotide biosynthesis axis and key metabolic changes in SCLC. In addition, we demonstrated that the RRM1 protein is essential for the formation of SCLC tumors, making it a feasible chemotherapeutic target for treating patients with SCLC. Indeed, several new RRM1 inhibitors have been investigated in clinical and pre-clinical phase trials. TAS1553, which is currently in a clinical trial for myeloid neoplasms, is a selective and orally available novel small molecule RNR inhibitor that abrogates protein–protein interactions between RNR subunits. Schlafen 11 (SLFN11) is an RNR inhibitor; its expression determines HU sensitivity, and it was identified as a consistent determinant of the response to poly (ADP-ribose) polymerase (PARP) inhibitors in SCLC^43,44^. Therefore, SLFN11 could be a prevalent biomarker for RRM1 or PARP inhibitors developed as molecular targeted therapies for SCLC, in addition to and independently of BRCA1/2 mutation and homologous recombination DNA pathway deficiency in pre-clinical models. Apoptosis of SCLC cells is an important area of research, but no conclusions have been reached yet. A previous study showed that high expression of the proapoptotic gene Bcl2-interacting mediator of cell death (BIM) predicts sensitivity to ABT-263, particularly in SCLC cell lines^45^. The responses in SCLC cells to the deletion of RRM1 differed between DMS 273 and H1048 cells. According to the results in Fig. 2, it seems that cell proliferation is suppressed in DMS 273 cells and apoptosis was induced in H1048 cells. However, as shown in Fig. 5, apoptosis-related genes were commonly activated in both DMS 273 and H1048 cells.

SCLC subtypes were characterized based on mRNA expression profiles defined by the differential expression of three key transcription regulators, achaete-scute homologue 1 (ASCL1; also known as ASH1), neurogenic differentiation factor 1 (NeuroD1), and POU class 2 homeobox 3 (POU2F3), and low expression of all three transcription factor signatures accompanied by an inflamed gene signature (SCLC-A, N, P, and I, respectively)^9^. The patterns of transcription factor programs and immune pathway activation define four major subtypes of SCLC with distinct therapeutic vulnerabilities^9^. When the expression of the three transcription factors was confirmed by referring to the public database COSMIC (Catalogue of Somatic Mutations in Cancer), the expression level of the three transcription factors was not high in DMS 273 cells, which could belong to SCLC-I; in H1048 cells, POU2F3 was expressed in this way, which could be classified as SCLC-P. This paper suggests that if RRM1 expression can be completely suppressed, tumor growth can be suppressed, but resistance by inhibitors is naturally assumed. Considering its drug resistance and that the combination of immunotherapy and chemotherapy is highly effective for SCLC-I^9^, the combination therapy of RRM1 inhibitor and immunotherapy appears to be the most effective at this stage. Single-cell level analysis is extremely important in considering treatment resistance^8^, and it is necessary to proceed with this analysis in the future.

## Methods

### Materials

Cell lines were purchased from the European Collection of Authenticated Cell Cultures (ECACC, Salisbury, UK), RIKEN Bio Resource Center (Tsukuba, Japan), and the American Type Culture Collection (ATCC, Manassas, VA, USA). RPMI 1640 (R8758) and phosphate-buffered saline (PBS) were purchased from Sigma-Aldrich (St. Louis, MO, USA). Fetal bovine serum (FBS) was purchased from Biowest (Nuaille, France). Dimethyl sulfoxide (DMSO) was purchased from FUJIFILM Wako Pure Chemicals (Osaka, Japan). COH29 was purchased from MedChemExpress (Monmouth Junction, NJ, USA). Triapine was purchased from Selleck (Houston, TX, USA). HU and gemcitabine were purchased from FUJIFILM Wako Pure Chemicals. Cell Counting Kit-8 was purchased from Dojindo Laboratories (Kumamoto, Japan). A Countess automated cell counter, including Trypan Blue and chamber slides, was purchased from Thermo Fisher Scientific (Waltham, MA, USA). Silencer Select Validated siRNA, negative control siRNA, Lipofectamine RNAiMAX reagent, and OPTI-MEM were purchased from Thermo Fisher Scientific. TRIzol Reagent and the SuperScript VILO cDNA synthesis kit were purchased from Thermo Fisher Scientific. TB Green Premix Ex Taq and synthesized primers were purchased from TaKaRa Bio (Shiga, Japan). Radioimmunoprecipitation assay buffer, TBS supplemented with 0.1% Tween-20 (TBS-T), and dry milk were purchased from Cell Signaling Technologies (Danvers, MA, USA). cOmplete Mini Protease Inhibitor Cocktail was purchased from Roche (Basel, Switzerland). Tris–glycine sodium dodecyl sulfate–polyacrylamide electrophoresis gels (4%–20%) and polyvinylidene difluoride membranes were purchased from Bio-Rad (Hercules, CA, USA). Primary antibodies specific for RRM1 (ab137114) were purchased from Abcam (Cambridge, UK). Primary antibodies specific for Chk1 (#2360), p-Chk1 (Ser-345, #2348), Chk2 (#3440), p-Chk2 (Thr-68, #2197), p53 (#2527), p-p53 (Ser-15, #9286), histone H2A (#12349), p-Histone H2A.X (Ser-139, #9718), and GAPDH (#2118) were purchased from Cell Signaling Technologies. Peroxidase-linked secondary antibodies for immunoblots, HRP-linked sheep anti-mouse IgG, and donkey anti-rabbit IgG were purchased from Cytiva (Malborough, MA, USA). The ECL Prime western blotting detection system was purchased from Cytiva. Nude mice were purchased from CLEA (Tokyo, Japan). AteloGene Local Use Quick Gelation was purchased from Koken (Tokyo, Japan). The FITC BrdU Flow Kit was purchased from Becton Dickinson (Franklin Lakes, NJ, USA). Mannitol, HPLC/MS-grade methanol, acetonitrile, and ammonium acetate were purchased from FUJIFILM Wako Pure Chemicals. InfinityLab Deactivator Additive (medronic acid) was purchased from Agilent Technologies Inc. (Santa Clara, CA). ADP, GDP, CDP, dADP, deoxy-GDP, and deoxy-CDP were purchased from Sigma-Aldrich. UDP was purchased from Jena Bioscience (Thuringia, Germany). Deoxy-UDP was purchased from MP Biomedicals (Santa Ana, CA). Methionine sulfone was purchased from Alfa Aesar (Lancashire, UK).

### Cell culture

Human SCLC DMS273 and NCI-H1048 cells were cultured in RPMI-1640 medium containing 10% FBS at 37 °C and 5% CO_2_. Cells were passaged every other day using 25 cm^2^ cell culture flasks and maintained in good condition.

### Cell survival and proliferation assays

DMS 273 and NCI-H1048 cells were seeded in 96-well cell culture plates containing RPMI 1640 with various concentrations of the following inhibitors: COH29, triapine, HU, and gemcitabine (FUJIFILM Wako Pure Chemicals). After incubation at 37 °C for 72 h, cell viability was analyzed using a WST-8 assay using Cell Counting Kit-8 (Dojindo).

For the proliferation assay, DMS273 and NCI-H1048 cells were transfected with siRNA for 24 and 48 h, harvested, and Trypan Blue-negative cells were counted using a Countess automated cell counter (Thermo Fisher Scientific) to indicate the number of viable cells.

### siRNA transfection

DMS273 and NCI-H1048 cells were cultured in 6-well plates until they reached 50%–60% confluence and then transfected with siRNAs or non-targeting control (final concentration 4 nM) using Lipofectamine RNAiMAX reagent (Thermo Fisher Scientific), according to the manufacturer’s instructions. The following siRNA sequences were used: siRRM1#1 sense 5′-GGA UCG CUG UCU CUA ACU UTT-3′ and antisense 5′-AAG UUA GAG ACA GCG AUC CTG-3′; siRRM1#2 sense 5′-CUA CAU UGC UGG ACU AAU TT-3′ and antisense 5′-AUU AGU CCC AGC AAU GUA GCT-3′; siRRM1#3 sense 5′-GCU GCA ACC UUG ACU ACU ATT-3′ and antisense 5′-UAG UAG UCA AGG UUG CAG CTG-3′. Negative control siRNA (NC) was obtained from Thermo Fisher Scientific. At 24 or 48 h after transfection, cells were harvested for qRT-PCR or western blot analyses.

### Quantitative RT-PCR

After cells had been washed with PBS, total RNA was isolated using TRIzol Reagent (Thermo Fisher Scientific) and complementary DNA (cDNA) was synthesized using a SuperScript VILO cDNA synthesis kit (Thermo Fisher Scientific). Real-time RT-PCR was carried out using specific primers (TaKaRa Bio) with a QuantStudio 3 system (Thermo Fisher Scientific). The fluorescence of the PCR products was monitored in real-time using TB Green Premix Ex Taq (TaKaRa Bio). Expression was calculating using the 2^−(Ct−Cc)^ method relative to that of the control, GAPDH.

### Western blotting

Cells were lysed in radioimmunoprecipitation assay buffer (150 mM NaCl, 1% Triton X-100, 0.5% sodium deoxycholate, 0.1% SDS, 50 mM Tris, pH 8.0) supplemented with cOmplete Mini Protease Inhibitor Cocktail (Roche, Switzerland) on ice for 10 min, sonicated, and centrifuged at 15,000 × *g* for 30 min. The protein content of the supernatant was measured using a BCA assay (Thermo Fisher Scientific). Identical amounts of protein samples were separated by 4%–20% SDS/PAGE and transferred to PVDF membranes, which were blocked for 1 h at room temperature with 5% milk in TBS-T (Cell Signaling Technologies). When the size of the protein was already known, the blots were cut prior to hybridization with primary antibodies. The blocked membrane was incubated with primary antibodies overnight at 4 °C (1:1,000 dilution), washed with TBS-T, and then incubated with horseradish peroxidase–conjugated secondary antibodies (1:10,000 dilution; Cytiva). Signals were developed using the ECL Prime western blotting detection system (Cytiva). Chemiluminescence signals were acquired and analyzed using a FUSION Chemiluminescence Imaging System (VILBER, Collégien, France). The images of all blots in this paper were created in compliance with the journal’s digital image and integrity policies. We performed densitometric analysis on all the immunoblots using ImageJ from the National Institutes of Health (Bethesda, MD, USA). The quantified results are included as a Supplementary Table S1. Densitometry data were normalized to the GAPDH level, which was the loading control for blots, or to the unphosphorylated protein level for phosphorylated protein. Because no image that shows the full-length blots was obtained, the original images and those with membrane edges visible are presented in the Supplementary Information. To show the edges of the membrane, images were processed using Adobe Photoshop (San Jose, CA, USA). The following primary antibodies were used in this study: anti-RRM1 (ab137114, Abcam), Chk1 (#2360, Cell Signaling Technologies), p-Chk1 (Ser-345, #2348, Cell Signaling Technologies), Chk2 (#3440, Cell Signaling Technologies), p-Chk2 (Thr-68, #2197, Cell Signaling Technologies), p53 (#2527, Cell Signaling Technologies), p-p53 (Ser-15, #9286, Cell Signaling Technologies), histone H2A (#12349, Cell Signaling Technologies), p-histone H2A.X (Ser-139, #9718, Cell Signaling Technologies), and GAPDH (#2118, Cell Signaling Technologies).

### Animal studies

All experimental nude mice were handled according to the institutional guidelines established by the Animal Care Committee of the National Cancer Center. The study protocols were approved by the Animal Ethics Committee of the National Cancer Center (approval number #T17502). We also confirmed that the mouse experiments were carried out in compliance with the ARRIVE guidelines^46,47^. To anesthetize the mice, we used a mixture of medetomidine (0.3 mg/kg), midazolam (4.0 mg/kg), and butorphanol (5.0 mg/kg) by intraperitoneal administration^48^. At 24 h after siRNA transfection, 1 × 10^7^ cells were suspended in 200 μL RPMI-1640 medium and injected subcutaneously into the flank of eight-week-old female nude mice (CLEA). In vivo siRNA transfection was then performed using AteloGene Local Use Quick Gelation (Koken) with anesthesia according to the manufacturer’s instructions. Briefly, 200 μL AteloGene containing siCont or si*RRM1* (2 nmol) was injected near the tumor every 3 days. The tumor volume was measured every 3 days and calculated as follows:

$$ {\text{tumor}}\;{\text{volume}} = \frac{{{\text{length}} \times {\text{width}}^{2} }}{2} $$

The photographs of gross pathology were taken with the digital camera COOLPIX W100 (Nikon, Tokyo, Japan) and images were processed using Adobe Photoshop. All histological assessments were performed on paraffin-embedded tissues obtained from the xenografted tumors in mice. Resected specimens were fixed in 10% formalin and embedded in paraffin, and hematoxylin and eosin staining was used for routine pathological examination. We prepared and used 4 μm-thick paraffin sections cut from a paraffin block that were histologically representative of the tumor. The procedure for histological assessment was previously described^49,50^. Images were acquired with a BZ-X710 All-in-One Fluorescence Microscope (Keyence, Osaka, Japan).

### Cell cycle analysis

The cell cycle distribution of transfected cells was analyzed using flow cytometry. Cells were fixed and stained using a BrdU Flow Kit (Becton Dickinson) according to the manufacturer’s instructions. At 24 h or 48 h after siRNA transfection, cells were collected, incubated with 10 μM 5-bromo-2’-deoxyuridine (BrdU) for 1 h, and then fixed and incubated for 30 min at 4 °C. BrdU was detected using anti-BrdU FITC. DNA content was measured using 7-amino-actinomycin D (7-AAD). Stained cells were analyzed using a Becton Dickinson FACSMelody instrument (Franklin Lakes, NJ, USA). FlowJo software (FlowJo, LLC, Ashland, OR, United States) was used to analyze data and generate graphs.

### Metabolite extraction

After the culture medium was aspirated, plated cells were washed twice with 5% mannitol solution (10 mL and then 2 mL), treated with 800 µL of methanol, and left at rest for 30 s to inactivate enzymes^35,51,52^. The cell extract was then treated with 550 µL of Milli-Q water containing internal standards (H3304-1002, HMT) and left at rest for a further 30 s. The extract was centrifuged at 2,300 × *g* and 4 °C for 5 min and then 800 µL of the upper aqueous layer was centrifugally filtered through a Millipore 5 kDa cutoff filter (UltrafreeMC-PLHCC, HMT) to remove macromolecules (9,100 × *g*, 4 °C, 120 min). The filtrate was centrifugally concentrated and re-suspended in 50 µL of Milli-Q water for metabolome analysis at HMT.

### Metabolome analysis

Metabolome analysis was conducted using the ω*-Scan* package (HMT) using a capillary electrophoresis Orbitrap mass spectrometer (CE-FTMS) based on previously described methods^35–38^. Briefly, CE-FTMS analysis was carried out using an Agilent CE capillary electrophoresis system (Agilent Technologies) equipped with an Orbitrap mass spectrometer, QExactive Plus (Thermo Fisher Scientific) that were controlled using ChemStation software version B.04.03 (Agilent Technologies) and Xcalibur software version 3.1.66.10 (Thermo Fisher Scientific), respectively. Metabolites were separated using a fused silica capillary (50 μm i.d. × 80 cm total length; Polymicro Technologies, Phoenix, AZ, USA) with commercial electrophoresis buffer (H3301-1001 and H3302-1021 for cation and anion analyses, respectively, HMT) as the electrolyte. MS spectra were scanned from *m/z* 70 to 1050 for anion analysis and from *m/z* 60 to 900 for cation analysis. Peaks were extracted using MasterHands automatic integration software (Keio University, Tsuruoka, Yamagata, Japan)^38^ to obtain peak information, including *m/z*, peak area, and migration time (MT). Signal peaks corresponding to isotopomers, adduct ions, and other ion products of known metabolites were excluded. The remaining peaks were annotated according to the HMT metabolite database based on their *m*/*z* values and MTs. The areas of the annotated peaks were normalized based on internal standards and sample amounts to obtain the relative levels of each metabolite.

### HPLC analysis

HPLC instrument modules were from the 1260 Infinity line from Agilent Technologies: binary pumps (Model G1312B), autosampler (G1367E), and temperature-controlled column compartment (Model G1316A). A stock solution of 100 mM ammonium acetate was made by dissolving 0.1 mol of ammonium acetate in water, adjusting to pH 9.0 with ammonium hydroxide, and correcting the final volume to 1 L with water. Hydrophilic interaction chromatography (HILIC) was performed using a 150 mm × 2.1 mm i.d. InfinityLab Poroshell 120 HILIC-Z column (Agilent Technologies). Solvent A was made by mixing 100 mL of the stock solution and 900 mL of water, which yielded a final concentration of 10 mM ammonium acetate (pH 9.0) in water. Solvent B was made by mixing 100 mL of the stock solution with 900 mL of acetonitrile, which yielded a final concentration of 10 mM ammonium acetate (pH 9.0) in 90% acetonitrile. The deactivator additive (5 mM methylenediphosphonic acid) was spiked into the solvents for a final concentration of 5 μM for analysis^53^. The flow rate was 0.3 mL/min, and the column temperature was set at 45 °C. A sample volume of 1 μL was injected into the column for each experiment. The gradient began initially at 95% B, decreasing linearly to 70% at 0.1 min, decreasing linearly again to 65% at 5.9 min, and subsequently re-equilibrating at 95% B for a further 6 min. The sample temperature was maintained at 4 °C in the autosampler prior to analysis. Mass spectrometer analysis was performed using an Agilent 6430 triple quadrupole mass spectrometer (Agilent Technologies) equipped with an ESI probe in negative-ion mode. A capillary voltage of − 3,500 V, a source temperature of 350 °C, gas flow of 10 L/min, and nebulizer of 40 psi were used. Raw data were sampled at 750 ms/cycle in Dynamic MRM mode. The concentrations of individual nucleotides were calculated from the peak area in the chromatogram detected with MRM relative to the internal standard, methionine sulfone.

### Statistical analyses

Unless otherwise indicated, data are reported as the mean ± S.D. Statistical differences among the groups were assessed by means of one-way analysis of variance for in vitro and in vivo data analysis. Adjusted *p* values were calculated using Dunnett’s multiple comparisons test and *p* values are indicated as *, < 0.05; **, < 0.01; and ***, < 0.005. For metabolomic data analysis, we used the Welch *t*-test and *p* values are indicated as *, < 0.05; **, < 0.01; and ***, < 0.001. Statistical analysis was done using Graph Pad Prism (GraphPad Software, Chicago, IL, USA).

## Supplementary Information

Supplementary Information 1.

Supplementary Information 2.

Supplementary Information 3.

## Data Availability

All data relevant to the study are included in this published article and related files.

## References

[1] Gazdar AF, Bunn PA, Minna JD (2017). Small-cell lung cancer: What we know, what we need to know and the path forward. Nat. Rev. Cancer.

[2] Poirier JT (2020). New approaches to SCLC therapy: from the laboratory to the clinic. J. Thorac. Oncol..

[3] Taniguchi H, Sen T, Rudin CM (2020). Targeted therapies and biomarkers in small cell lung cancer. Front Oncol..

[4] Ko J, Winslow MM, Sage J (2020). Mechanisms of small cell lung cancer metastasis. EMBO Mol. Med..

[5] Singh S (2020). FDA approval summary: lurbinectedin for the treatment of metastatic small cell lung cancer. Clin. Cancer Res..

[6] Rudin CM (2019). Molecular subtypes of small cell lung cancer: a synthesis of human and mouse model data. Nat. Rev. Cancer.

[7] Byers LA (2012). Proteomic profiling identifies dysregulated pathways in small cell lung cancer and novel therapeutic targets including PARP1. Cancer Discov..

[8] Stewart CA (2020). Single-cell analyses reveal increased intratumoral heterogeneity after the onset of therapy resistance in small-cell lung cancer. Nat. Cancer.

[9] Gay CM (2021). Patterns of transcription factor programs and immune pathway activation define four major subtypes of SCLC with distinct therapeutic vulnerabilities. Cancer Cell.

[10] Huang F (2018). Inosine monophosphate dehydrogenase dependence in a subset of small cell lung cancers. Cell Metab..

[11] Makinoshima H (2018). Metabolic determinants of sensitivity to phosphatidylinositol 3-kinase pathway inhibitor in small-cell lung carcinoma. Cancer Res..

[12] Kodama M (2020). A shift in glutamine nitrogen metabolism contributes to the malignant progression of cancer. Nat. Commun..

[13] D'Angiolella V (2012). Cyclin F-mediated degradation of ribonucleotide reductase M2 controls genome integrity and DNA repair. Cell.

[14] Guarino E, Salguero I, Kearsey SE (2014). Cellular regulation of ribonucleotide reductase in eukaryotes. Semin. Cell Dev. Biol..

[15] Hakansson P, Hofer A, Thelander L (2006). Regulation of mammalian ribonucleotide reduction and dNTP pools after DNA damage and in resting cells. J. Biol. Chem..

[16] Kumar D, Viberg J, Nilsson AK, Chabes A (2010). Highly mutagenic and severely imbalanced dNTP pools can escape detection by the S-phase checkpoint. Nucleic Acids Res..

[17] Mathews CK (2019). Deoxyribonucleotide salvage falls short in whole animals. J. Biol. Chem..

[18] Mathews CK (2006). DNA precursor metabolism and genomic stability. FASEB J..

[19] Lin S (2019). The mitochondrial deoxyguanosine kinase is required for cancer cell stemness in lung adenocarcinoma. EMBO Mol. Med..

[20] Tran P (2019). De novo dNTP production is essential for normal postnatal murine heart development. J. Biol. Chem..

[21] Nordlund P, Reichard P (2006). Ribonucleotide reductases. Annu. Rev. Biochem..

[22] Huang F (2021). Guanosine triphosphate links MYC-dependent metabolic and ribosome programs in small-cell lung cancer. J. Clin. Invest..

[23] Aye Y, Li M, Long MJ, Weiss RS (2015). Ribonucleotide reductase and cancer: biological mechanisms and targeted therapies. Oncogene.

[24] Chiappori AA (2010). Features of potentially predictive biomarkers of chemotherapeutic efficacy in small cell lung cancer. J. Thorac. Oncol..

[25] Ceppi P (2008). Excision repair cross complementing-1 and topoisomerase IIalpha gene expression in small-cell lung cancer patients treated with platinum and etoposide: a retrospective study. J. Thorac. Oncol..

[26] Shimizu J (2008). mRNA expression of RRM1, ERCC1 and ERCC2 is not associated with chemosensitivity to cisplatin, carboplatin and gemcitabine in human lung cancer cell lines. Respirology.

[27] Wonganan P (2012). Silencing of ribonucleotide reductase subunit M1 potentiates the antitumor activity of gemcitabine in resistant cancer cells. Cancer Biol. Ther..

[28] Kim SH (2014). RRM1 maintains centrosomal integrity via CHK1 and CDK1 signaling during replication stress. Cancer Lett..

[29] Tokunaga Y (2015). Potent effect of adenoviral vector expressing short hairpin RNA targeting ribonucleotide reductase large subunit M1 on cell viability and chemotherapeutic sensitivity to gemcitabine in non-small cell lung cancer cells. Eur. J. Cancer.

[30] Sagawa M (2017). Ribonucleotide reductase catalytic subunit M1 (RRM1) as a novel therapeutic target in multiple myeloma. Clin. Cancer Res..

[31] Chen G (2019). Acetylation regulates ribonucleotide reductase activity and cancer cell growth. Nat. Commun..

[32] Shu Z (2020). Cell-cycle-dependent phosphorylation of RRM1 ensures efficient DNA replication and regulates cancer vulnerability to ATR inhibition. Oncogene.

[33] Postel-Vinay S (2012). The potential of exploiting DNA-repair defects for optimizing lung cancer treatment. Nat. Rev. Clin. Oncol..

[34] Warren NJH, Eastman A (2020). Comparison of the different mechanisms of cytotoxicity induced by checkpoint kinase I inhibitors when used as single agents or in combination with DNA damage. Oncogene.

[35] Maruyama A (2019). Extraction of aqueous metabolites from cultured adherent cells for metabolomic analysis by capillary electrophoresis-mass spectrometry. J. Vis. Exp..

[36] Ohashi Y (2008). Depiction of metabolome changes in histidine-starved *Escherichia coli* by CE-TOFMS. Mol. Biosyst..

[37] Sasaki K (2019). Metabolomics platform with capillary electrophoresis coupled with high-resolution mass spectrometry for plasma analysis. Anal. Chem..

[38] Sugimoto M, Wong DT, Hirayama A, Soga T, Tomita M (2010). Capillary electrophoresis mass spectrometry-based saliva metabolomics identified oral, breast and pancreatic cancer-specific profiles. Metabolomics.

[39] Gautam A, Li ZR, Bepler G (2003). RRM1-induced metastasis suppression through PTEN-regulated pathways. Oncogene.

[40] Fan H, Huang A, Villegas C, Wright JA (1997). The R1 component of mammalian ribonucleotide reductase has malignancy-suppressing activity as demonstrated by gene transfer experiments. Proc. Natl. Acad. Sci. U S A.

[41] Shivapurkar N, Reddy J, Chaudhary PM, Gazdar AF (2003). Apoptosis and lung cancer: a review. J. Cell Biochem..

[42] Travis WD (2012). Update on small cell carcinoma and its differentiation from squamous cell carcinoma and other non-small cell carcinomas. Mod. Pathol..

[43] Murai J (2016). Resistance to PARP inhibitors by SLFN11 inactivation can be overcome by ATR inhibition. Oncotarget.

[44] Murai J, Thomas A, Miettinen M, Pommier Y (2019). Schlafen 11 (SLFN11), a restriction factor for replicative stress induced by DNA-targeting anti-cancer therapies. Pharmacol. Ther..

[45] Faber AC (2015). Assessment of ABT-263 activity across a cancer cell line collection leads to a potent combination therapy for small-cell lung cancer. Proc. Natl. Acad. Sci. U S A.

[46] Festing MF, Altman DG (2002). Guidelines for the design and statistical analysis of experiments using laboratory animals. ILAR J..

[47] Kilkenny C, Browne WJ, Cuthill IC, Emerson M, Altman DG (2010). Improving bioscience research reporting: the ARRIVE guidelines for reporting animal research. PLoS Biol..

[48] Kawai S, Takagi Y, Kaneko S, Kurosawa T (2011). Effect of three types of mixed anesthetic agents alternate to ketamine in mice. Exp. Anim..

[49] Makinoshima H (2012). PTPRZ1 regulates calmodulin phosphorylation and tumor progression in small-cell lung carcinoma. BMC Cancer.

[50] Suzuki M (2013). Identification of a lung adenocarcinoma cell line with CCDC6-RET fusion gene and the effect of RET inhibitors in vitro and in vivo. Cancer Sci..

[51] Makinoshima H (2014). Epidermal growth factor receptor (EGFR) signaling regulates global metabolic pathways in EGFR-mutated lung adenocarcinoma. J. Biol. Chem..

[52] Makinoshima H (2015). Signaling through the phosphatidylinositol 3-kinase (PI3K)/mammalian target of rapamycin (mTOR) axis is responsible for aerobic glycolysis mediated by glucose transporter in epidermal growth factor receptor (EGFR)-mutated lung adenocarcinoma. J. Biol. Chem..

[53] Hsiao JJ, Potter OG, Chu TW, Yin H (2018). Improved LC/MS methods for the analysis of metal-sensitive analytes using medronic acid as a mobile phase additive. Anal. Chem..

