# Supplementary material for: De novo deoxyribonucleotide biosynthesis regulates cell growth and tumor progression in small-cell lung carcinoma
Source: Sci Rep. 2021 Jun 29;11:13474. doi: 10.1038/s41598-021-92948-9 (PMC8242079; doi:10.1038/s41598-021-92948-9)
Supplement: Supplementary file 1 — Supplementary Information 1. [file 41598_2021_92948_MOESM1_ESM.pdf]

Supplementary Information

De novo deoxyribonucleotide biosynthesis regulates cell growth and tumor progression in small-cell lung carcinoma

Ami Maruyama, Yuzo Sato, Joji Nakayama, Junko Murai, Takamasa Ishikawa, Tomoyoshi Soga, Hideki Makinoshima

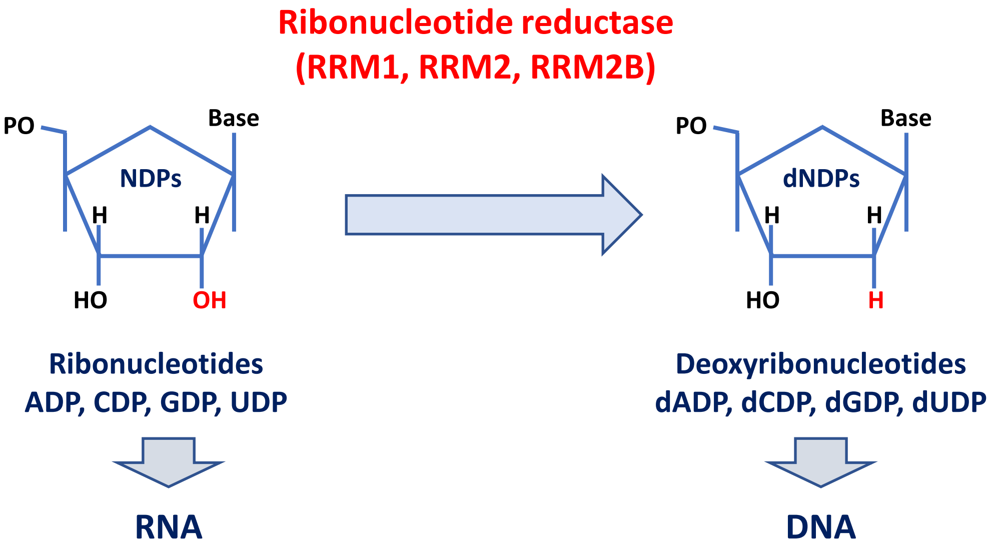

Figure. S1 Schematic diagram of reaction catalyzed by ribonucleotide reductase.

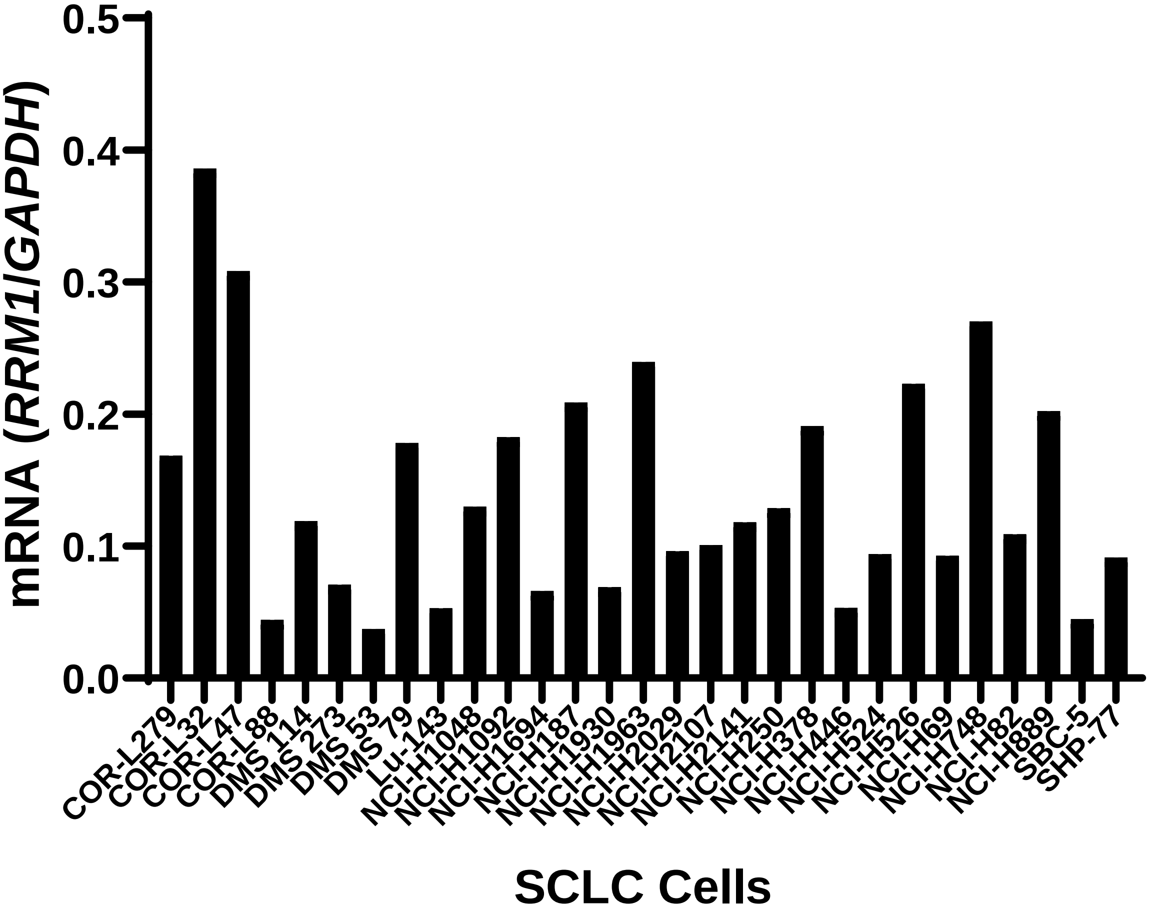

Figure. S2 Relative level of *RRM1* mRNAs in SCLC cells.
Total RNA was isolated from SCLC cells. Relative mRNA level od *RRM1* to *GAPDH* was shown. Data are shown as the mean ± SD. (*n* = 3).

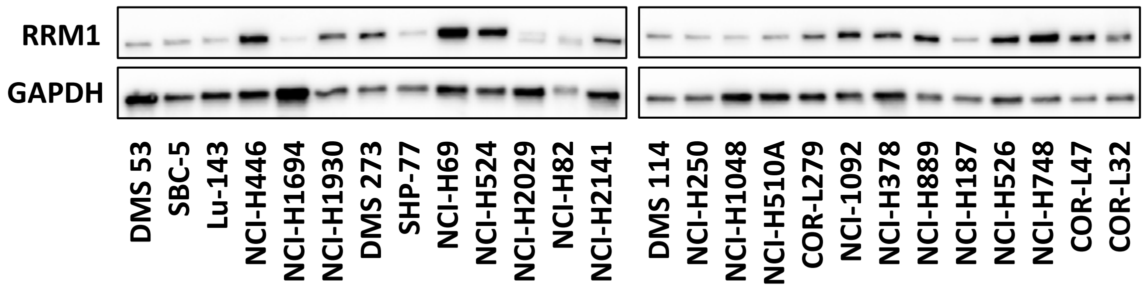

Supplementary Fig. S3 RRM1 Protein level in SCLC cells.
Western blot analysis of RRM1 expression in 26 SCLC cell lines. GAPDH was loading control. The blots were cropped and full-length blots are included in a Supplementary Information file.

A

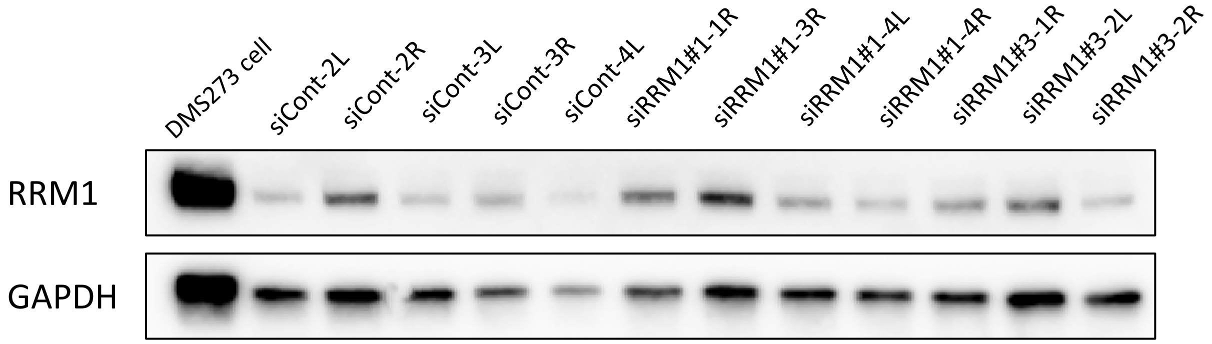

B

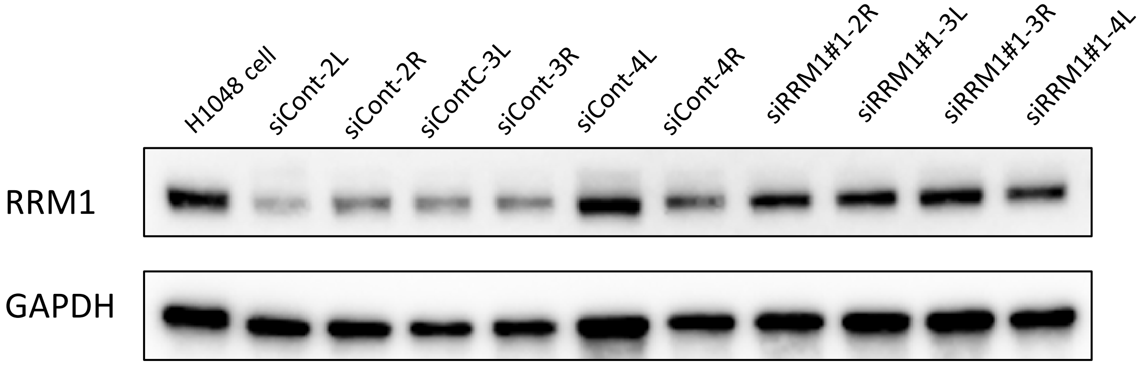

Supplementary Fig. S4 Western blot analysis of RRM1 expression in mouse-xenografted tumours.
DMS 273 tumors (*A*) and H1048 tumors (*B*) were shown. GAPDH was loading control. The blots were cropped and full-length blots are included in a Supplementary Information file.

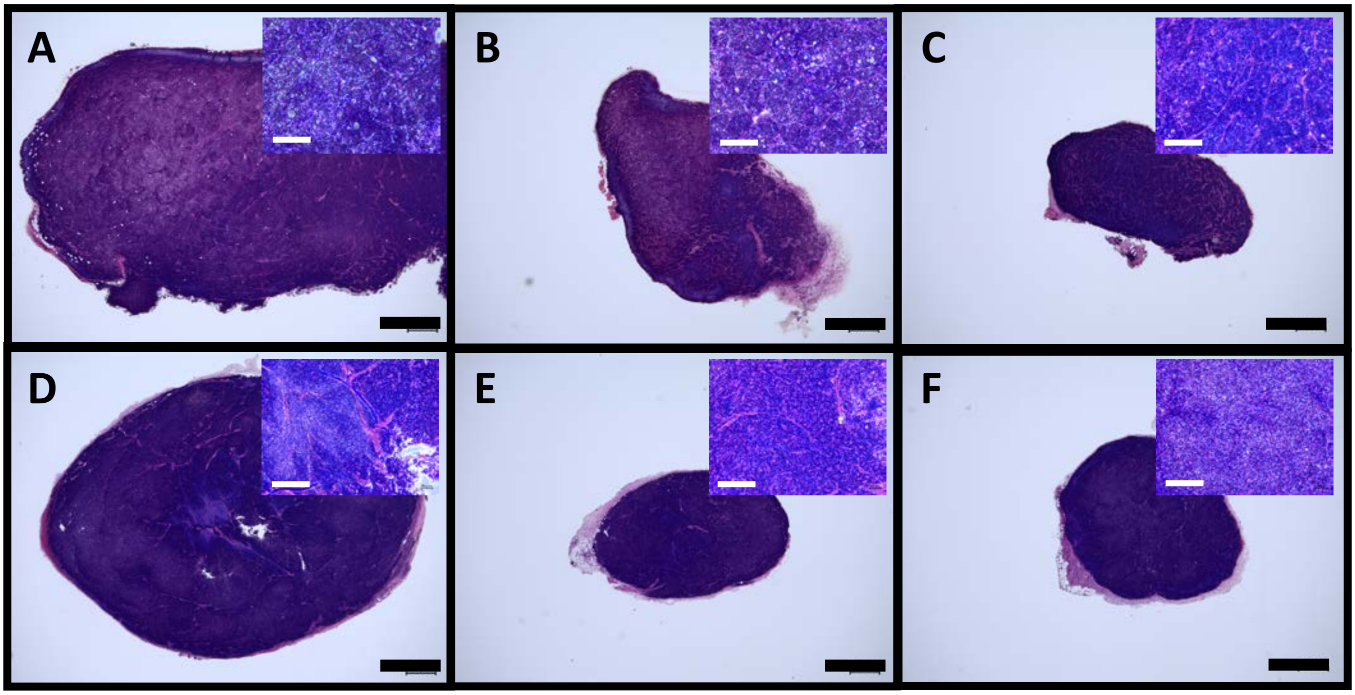

Supplementary Fig. S5 Histological characterization of the SCLC xenografts. Histological features of the xenograft. For viewing cellular and tissue structure detail, hematoxylin and eosin staining were used. DMS 273 tumors (*A*-*C*) and H1048 tumors (*D*-*F*) were shown. Images were acquired with BZ-X710 All-in-One Fluorescence Microscope (Keyence, Osaka, Japan). Black scale bars were 1,000 μm and white scale bars were 100 μm.

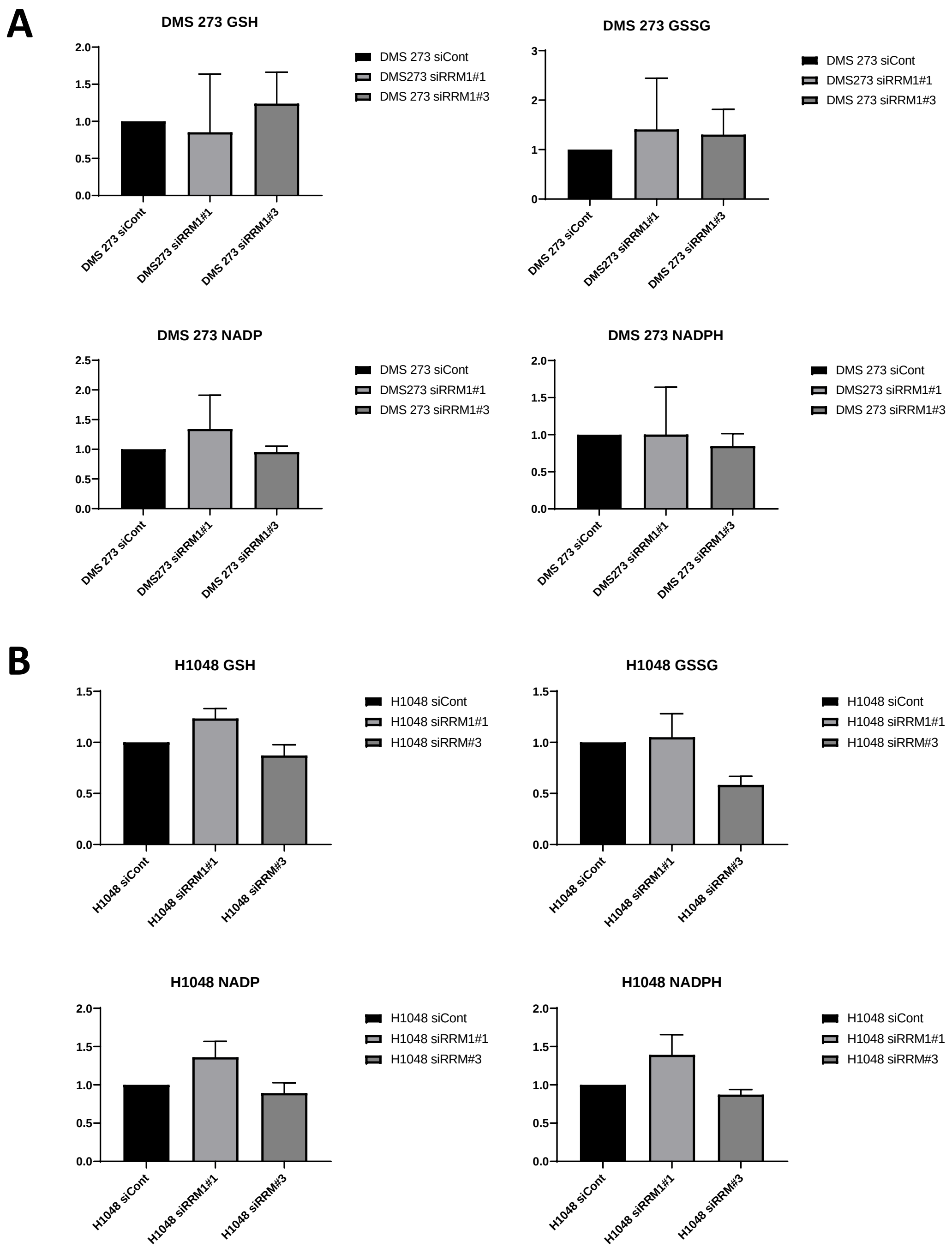

Supplementary Fig. S6 Metabolome analysis oxidative stress marker.
Relative intracellular levels of key metabolites in the nucleotide biosynthesis pathway after RRM1 inhibition in DMS 273 (*A*) and H1048 (*B*) cells. Other metabolites are listed in Supplementary Table 1. Data represent the mean ± SD (*n* = 3).

Original images of gross pathology

Figure 3E

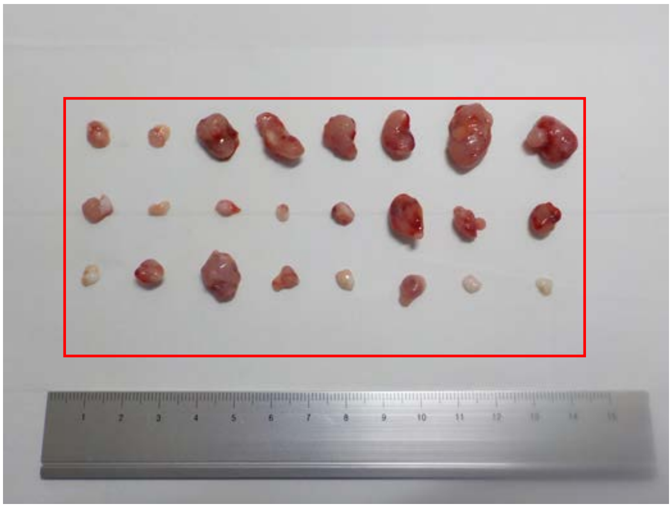

Figure 3F

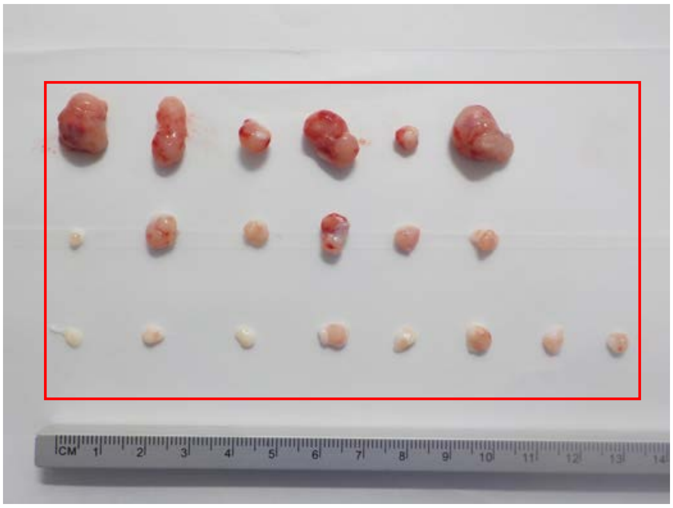

Original images of blots

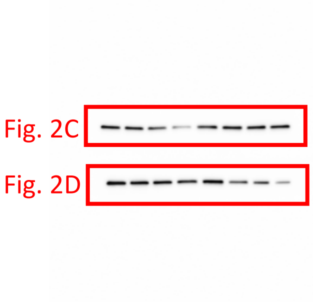

Figure 2
GAPDH

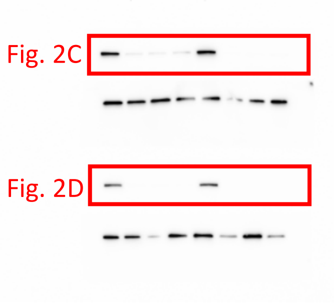

Figure 2
RRM1

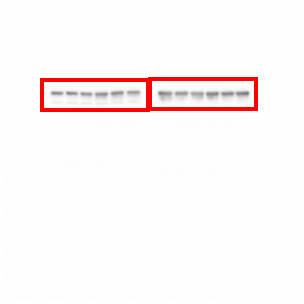

Figure 5
GAPDH

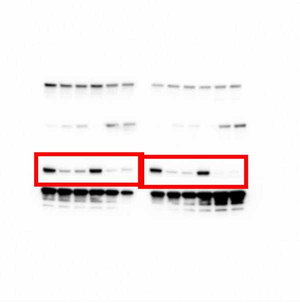

Figure 5
RRM1

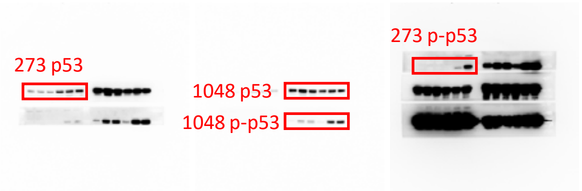

Figure 5
P53 & p-p53

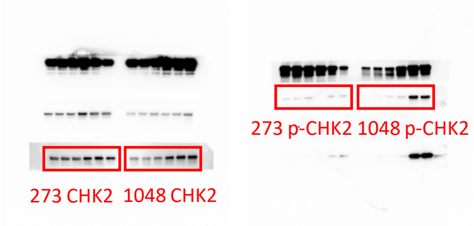

Figure 5
CHK2 & p-CHK2

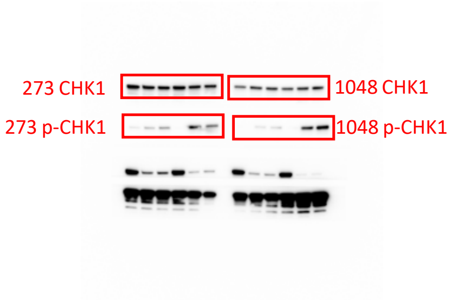

Figure 5
CHK1 & p-CHK1

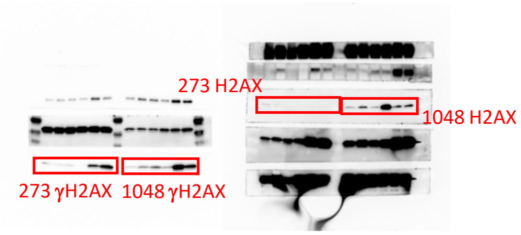

Figure 5
H2AX & γH2AX

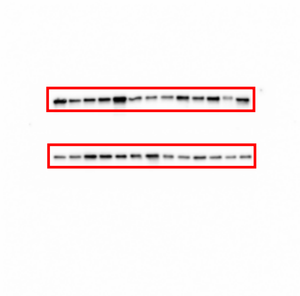

Supplementary Fig S3
GAPDH

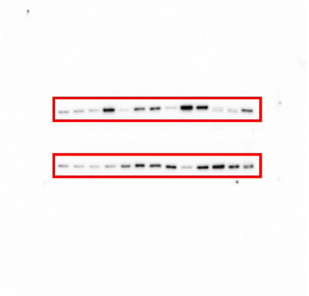

Supplementary Fig S3
RRM1

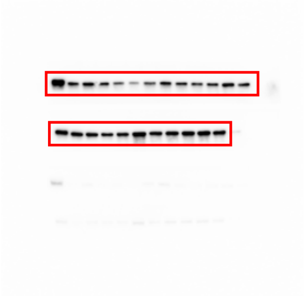

Supplementary Fig S4
GAPDH

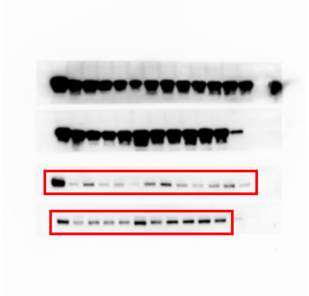

Supplementary Fig S4
RRM1

Membrane edges of blots

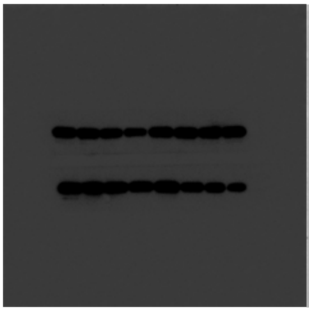

Figure 2
GAPDH

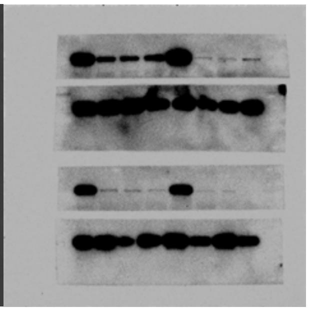

Figure 2
RRM1

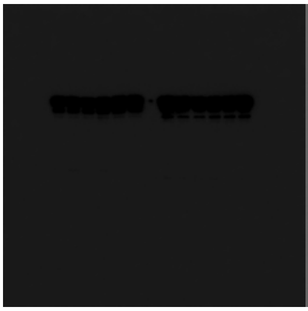

Figure 5
GAPDH

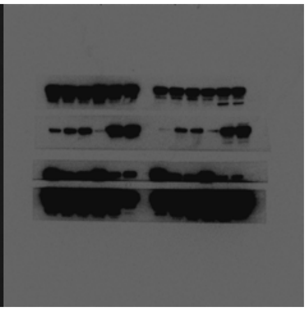

Figure 5
RRM1

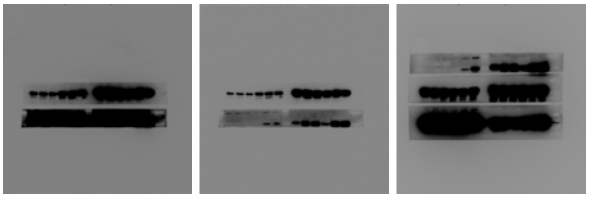

Figure 5
P53 & p-p53

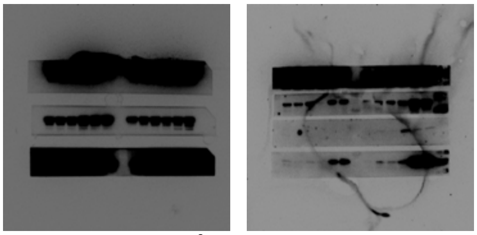

Figure 5
CHK2 & p-CHK2

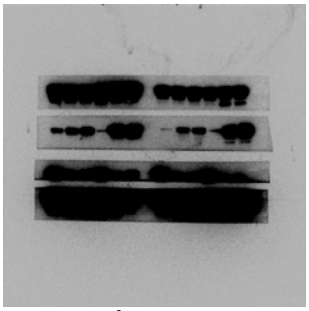

Figure 5
CHK1 & p-CHK1

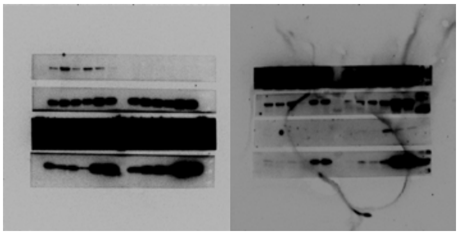

Figure 5
H2AX & γH2AX

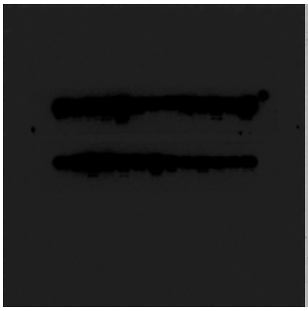

Supplementary Fig S3
GAPDH

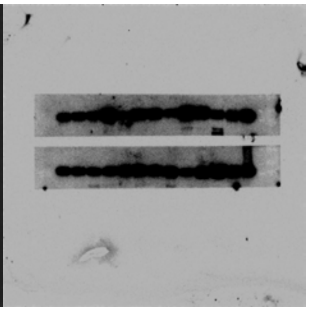

Supplementary Fig S3
RRM1

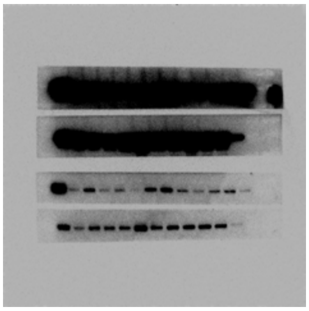

Supplementary Fig S4
GAPDH & RRM1
